# Aging and Neurodegeneration: New Approaches to Neurodegeneration and Aging

**DOI:** 10.1093/geroni/igaa057.2631

**Published:** 2020-12-16

**Authors:** Nancy Bonini

## Abstract

Model organisms like C. elegans and Drosophila are powerful systems to help dissect molecular genetic aspects of complex biological processes. Here we will present a number of biological questions and approaches that have been used to study questions of neural integrity and healthful aging using these systems.

